# Flow Cytofluorimetric Analysis of Anti-LRP4 (LDL Receptor-Related Protein 4) Autoantibodies in Italian Patients with Myasthenia Gravis

**DOI:** 10.1371/journal.pone.0135378

**Published:** 2015-08-18

**Authors:** Mariapaola Marino, Flavia Scuderi, Daniela Samengo, Giorgia Saltelli, Maria Teresa Maiuri, Chengyong Shen, Lin Mei, Mario Sabatelli, Giovambattista Pani, Giovanni Antonini, Amelia Evoli, Emanuela Bartoccioni

**Affiliations:** 1 Institute of General Pathology, School of Medicine, Università Cattolica S. Cuore, Rome, Italy; 2 Department of Laboratory Medicine, School of Medicine, Università Cattolica S. Cuore, Rome, Italy; 3 Department of Neurology, Mental Health and Sensory Organs (NESMOS), Faculty of Medicine and Psychology, University of Rome “Sapienza”, Rome, Italy; 4 Department of Neuroscience and Regenerative Medicine and Department of Neurology, Medical College of Georgia, Georgia Regents University, Augusta, Georgia, United States of America; 5 Centro Clinico NEMO-Roma, Policlinico A. Gemelli, Rome, Italy; 6 Department of Neuroscience, School of Medicine, Università Cattolica S. Cuore, Rome, Italy; Istanbul University, TURKEY

## Abstract

**Background:**

Myasthenia gravis (MG) is an autoimmune disease in which 90% of patients have autoantibodies against the muscle nicotinic acetylcholine receptor (AChR), while autoantibodies to muscle-specific tyrosine kinase (MuSK) have been detected in half (5%) of the remaining 10%. Recently, the low-density lipoprotein receptor-related protein 4 (LRP4), identified as the agrin receptor, has been recognized as a third autoimmune target in a significant portion of the double sero-negative (dSN) myasthenic individuals, with variable frequency depending on different methods and origin countries of the tested population. There is also convincing experimental evidence that anti-LRP4 autoantibodies may cause MG.

**Methods:**

The aim of this study was to test the presence and diagnostic significance of anti-LRP4 autoantibodies in an Italian population of 101 myasthenic patients (55 dSN, 23 AChR positive and 23 MuSK positive), 45 healthy blood donors and 40 patients with other neurological diseases as controls. All sera were analyzed by a cell-based antigen assay employing LRP4-transfected HEK293T cells, along with a flow cytofluorimetric detection system.

**Results:**

We found a 14.5% (8/55) frequency of positivity in the dSN-MG group and a 13% frequency of co-occurrence (3/23) in both AChR and MuSK positive patients; moreover, we report a younger female prevalence with a mild form of disease in LRP4-positive dSN-MG individuals.

**Conclusion:**

Our data confirm LRP4 as a new autoimmune target, supporting the value of including anti-LRP4 antibodies in further studies on Myasthenia gravis.

## Introduction

Myasthenia gravis (MG) is a disorder of neuromuscular transmission characterized by fluctuating muscle weakness and abnormal fatigability. Apart from rare cases of genetically determined myasthenic syndromes, the majority (up to 85%) of patients have auto-antibodies (auto-abs) directed against the nicotinic acetylcholine receptor (AChR) [1,2]; low affinity abs against AChR have been found in 5% of the remaining MG patients [3,4]; up to 50% of patients without anti-AChR abs display immunoreactivity to muscle-specific tyrosine kinase (MuSK) [5–7].

Both target antigens are membrane proteins that play essential roles at the neuromuscular junction (NMJ): the high concentration of AChRs at the top of postsynaptic folds is crucial for an efficient signal transmission from nerve to muscle. On the other hand, MuSK is essential for formation, maintenance, and regeneration of postsynaptic specializations, including AChR clustering [8]: neuronally-released agrin binds to the low-density lipoprotein receptor-related protein-4 (LRP4) and forms a complex that, in turn, activates MuSK [9,10]. LRP4 is located at the postsynaptic membrane of the NMJ and also on motor neurons in the brain and spinal cord [11–13].

Considering its critical role in AChR clustering, its large extracellular domain and the spatial proximity with MuSK, LRP4 was proposed as a possible autoantigen in patients with MG without detectable antibodies to previously identified components of the NMJ [14]. In fact, a proportion of patients without anti-AChR or anti-MuSK abs, and therefore classified as double-seronegative (dSN-MG), was found to harbor abs against LRP4 [15–19].

While anti-AChR abs accelerate degradation and activate complement-mediated destruction of the postsynaptic membrane, anti-MuSK abs appear to interfere with MuSK signaling and cause fragmentation of AChR clusters [20,21]. Further studies also indicate that anti-MuSK abs block the binding of the collagenic tail of acetylcholinesterase (AChE) to MuSK [22] and, accordingly, anti-AChE abs have been detected in patients with the pure ocular form of MG [23]. Even though LRP4 (along with MuSK) is not directly involved in neuromuscular transmission, there are convincing evidences that anti-LRP4 abs are pathogenic for MG. Schen and coworkers demonstrated that active immunization with the extracellular domain of LRP4 or passive transfer of IgGs purified from LRP4-immunized rabbits induced MG-associated symptoms and compromised neuromuscular transmission in mice. This effect was probably achieved thorough decreased cell surface LRP4 levels, inhibition of agrin-induced MuSK activation and AChR clustering and complement activation [24]. Very recently, Barik and coworkers showed that LRP4 ablation in mice led to loss of synaptic agrin, suggesting that LRP4 is essential to maintain the structural and functional integrity of the NMJ through the regulation of synaptic agrin stability [25]. Taken together, these evidences support the view that NMJ development during synaptogenesis, as its plasticity or function in adulthood, requires ongoing MuSK activation following agrin binding to LRP4.

Since diagnosis and management for dSN-MG patients can be puzzling, ascertaining the presence of autoantibodies against alternative protein targets could be of help in orienting clinical decisions.

In this study we tested sera from 101 MG Italian patients and from 45 healthy blood donors (HBD) and 40 patients with other neurological diseases (OND) as controls, employing a cell-based antigen assay (CBA) along with a cytofluorimetric detection system. Our data confirm LRP4 as a novel auto-antigen in MG patients; moreover, our results indicate that autoantibodies against LRP4 may coexist with anti-AChR and anti-MuSK abs suggesting new perspectives in MG-patients management.

## Materials and Methods

### Sera from MG patients and controls

The study included 101 Italian myasthenic patients seen at two distinct Hospitals in Rome (Gemelli Hospital at “Università Cattolica”, Department of Neuroscience, and S. Andrea Hospital at “La Sapienza” University, Neuromuscular Diseases Unit). All clinical investigations have been conducted according to the principles expressed in the Declaration of Helsinki.

Samples were collected according to the guidelines of a research study approved from the “Comitato Etico dell’Università Cattolica del Sacro Cuore” (Catholic University Ethical Committee; P/529/CE/2011) with written informed consent. All samples were anonymized. The participant consents were recorded in Catholic University laboratories.

All patients had generalized MG, as defined by the Myasthenia Gravis Foundation of America (MGFA) classification [26] (i.e. a disease affecting muscles distinct from ocular muscles, although ocular muscle weakness could be associated).

Anti-AChR and anti-MuSK abs were tested on the basis of standard assay (radioimmunoprecipitation using ^125^I α-bungarotoxin-AChR complexes or ^125^I-labeled recombinant human MuSK protein, respectively). We screened for anti-LRP4 immunoreactivity 55 sera from dSN-patients, 23 sera from AChR-positive and 23 from MuSK-positive patients. Sera from 45 HBD were analyzed as controls together with 40 OND: 11 polymyositis (PM), 10 mitochondrial myopathies (MM), 9 multiple sclerosis (MS) and 10 amyotrophic lateral sclerosis (ALS).

### Cell culture and transfection

Mammalian HEK293T cells (ATCC CRL-3216) were cultured in DMEM (EuroClone) plus 10% FCS (Sigma-Aldrich), 1% antibiotics, 1% glutamine (Sigma-Aldrich), 1% non-essential aminoacids (Sigma-Aldrich), 1 mM sodium pyruvate (Sigma-Aldrich) and maintained in a humidified incubator at 37°C with 5% CO_2_.

Constructs encoding full-length (fl) rat LRP4 and extracellular domain (ecto)-LRP4 in pcDNA3.1-Myc/His were prepared as previously described [9].

1μg of pcDNA3.1-LRP4fl-myc/His or 1μg of pcDNA3.1-LRP4ecto-myc/His were stably transfected in HEK293T cells (3 x 10^5^ in six-well plates) using the EFFECTENE reagent (Qiagen) according to manufacturer’s recommendations. We exposed un-transfected HEK293T cells to different concentrations of neomycin (G418; Invitrogen) and identified 1,5 mg/ml as the lethal dose, to which LRP4-transfected cells still survived after at least one week of exposure. Then, we checked the expression of LRP4 testing the transfected cells with control anti-LRP4 antiserum in cytofluorimetric assay. Instead, average transfection efficiency in LRP4fl-transfected cells (30–35%) was determined using an equal amount of a plasmid encoding the green fluorescent protein under the cytomegalovirus promoter (CMV-EGFP) and the percentage of fluorescent cells was determined 48 h after transfection by flow cytometry. To confirm the transfection efficacy, we performed indirect immunofluorescence on transfected and untransfected HEK293T cells. Briefly, cells were fixed with methanol, washed three times with cold PBS, blocked for 10 min at room temperature with PBS/BSA 5% and incubated for 30 min at 4°C with serum samples from patients and healthy controls, at 1:100 dilution in PBS/BSA 0.1% (PBS-BSA). FITC conjugated anti-human IgG goat antiserum (AXA Diagnostics, Italy) were added at a 1:100 dilution in PBS-BSA and the samples incubated for 30 min at 4°C. Cells were examined on a Nikon Eclipse E600 fluorescence microscope. A representative immunofluorescence is now displayed in S1 Fig, as additional material, which clearly shows much stronger positivity of transfected cells compared to untransfected controls when challenged with a previously validated anti-LPRP4 positive serum.

Similarly, transgene expression in LRP4ecto-transfected cells was verified by anti-Myc tag western-blotting after at least 48 h from transfection. Having verified that LRP4 transfection and selection had been successful, G418 concentration was reduced to 0,5–0,4 mg/ml; interestingly, in this setting the expression of LRP4 was stable for no more than two weeks, likely due to intrinsic toxicity of the gene product and selective overgrowth of LRP4 revertants or untransfected cells.

### Flow cytofluorimetric analysis

Fluorescence activated cell sorting (FACS) analysis was performed to detect binding of patients’ IgG to the surface of LRP4fl-HEK293T and of parental untransfected HEK293T (that do not express detectable levels of LRP4) cells as control, as previously described [27], with minor modifications. We initially tested twenty sera on both mock-transfected and untransfected cells, obtaining identical results. Based on this evidence, we kept on using untransfected cells for the rest of the study.

Briefly, cells (1 x 10^5^ per test) were resuspended in 50 μl PBS/BSA 0,1% (FACS buffer) and incubated with primary antibodies: sera from MG patients and HBD (dilution 1:5), or a commercially available rabbit anti-LRP4 antiserum (Atlas Antibodies, Stockholm, Sweden; dilution 1:150); the latter antibody, that served as positive control, is specific for LRP4 and does not cross-react with other members of the LRP protein family. Subsequently cell samples were stained using the appropriate Alexa-conjugated goat anti-human IgG or goat anti-rabbit secondary antibody (594 and 488 respectively, dilution 1:100). Cells were then analyzed on a FACScan (EpicsXL-MCL Coulter, Fullerton, California).

For each serum, the mean fluorescence intensity value (mean-F) on the LRP4fl-HEK293T cells was compared to unspecific binding determined on the parental HEK293T cell population to evaluate the antigen-specific immunoreactivity: positivity cut-off (1.5) was determined as the mean + 2.5 SD of the ratios between the LRP4fl-mean-F and the corresponding parental-mean-F of HBD sera (mean 1.1; SD 0.17). Each serum was tested at least two times with different cellular transfections. The rabbit Anti-LRP4 antiserum was assayed in each test session as quality control.

### Immunoprecipitation and Blotting

LRP4ecto-HEK293T cells (transfected with the Myc-tagged LRP4 ectodomain-cDNA) were employed to obtain a supernatant enriched of secreted soluble extracellular-LRP4. 48 hours (h) after transfection, cells were serum-starved for 24 h before collection of supernatants. Aliquots of one milliliter of supernatant were immunoprecipitated with 60 μl of human sera (mixtures of equal volumes of two or more patients’/HBD controls’ sera) and 20 μl of protein A-Sepharose for 3 h at 4°C, with continuous shaking. Immune-complexes were collected, washed three times with PBS-Triton 1%, dissolved in Laemmli sample buffer 6x, separated by SDS-PAGE, and transferred onto nitrocellulose (BioRad). Immunoblots were probed first with anti-c-Myc mAb (clone 9E10, Sc-40; Santa Cruz Biotechnology) (0,2 μg/ml), followed by horseradish peroxidase-conjugated secondary reagents (anti-mouse IgG, Amersham Biosciences, Inc.) (1:5000). Aliquots of 500 μl of supernatants from LRP4ecto-HEK293T cells and from EGFP-HEK293T cells (transfected with a plasmid encoding the green fluorescent-not secreted- protein), as positive and negative control respectively, were treated with a methanol/chloroform protocol [28] to recover proteins in dilute solutions. The protein concentrates were dissolved in Laemmli sample buffer and subjected to SDS-PAGE and blotted alongside with immunoprecipitates. The Quantity-One software (Bio-Rad Laboratories) was used for image documentation and analysis.

## Results

### Production of recombinant LRP4

**Fig 1:**
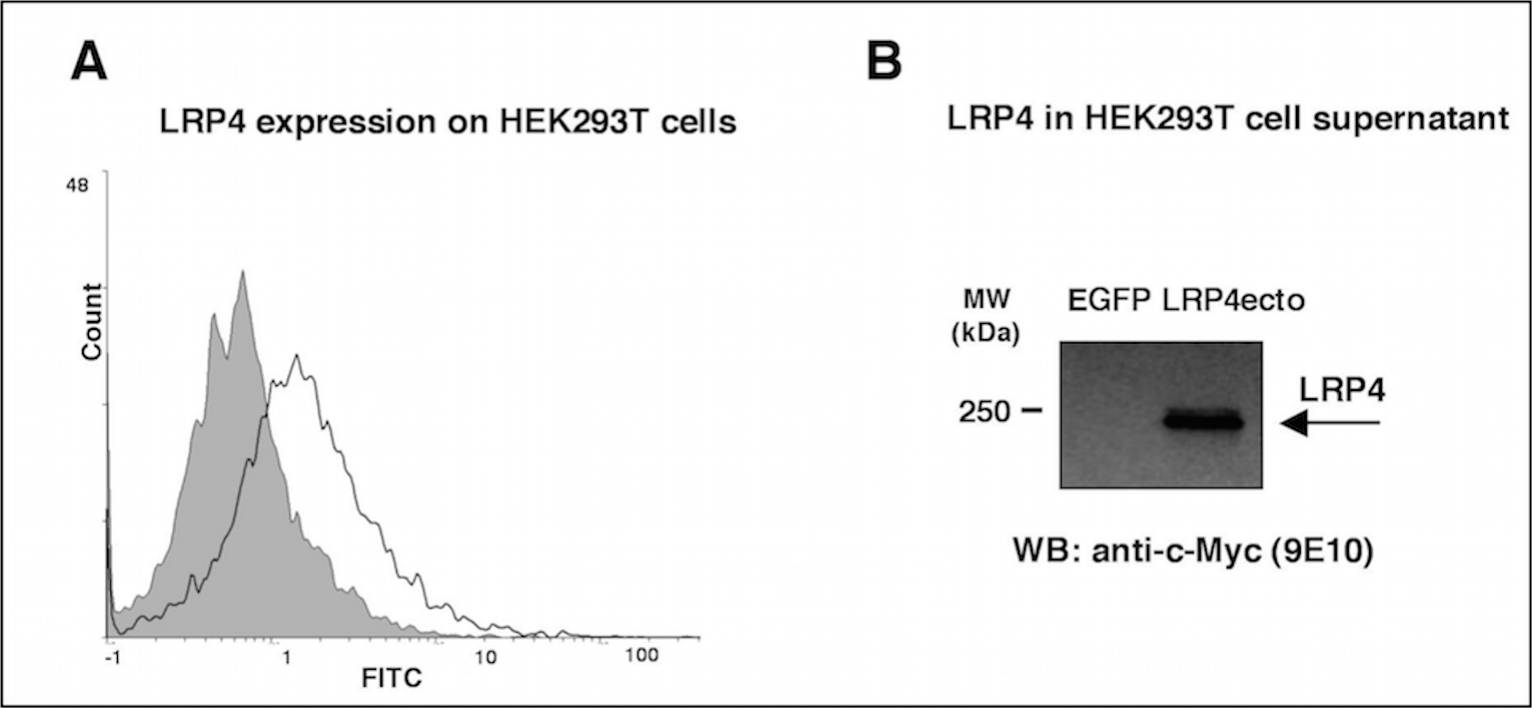
Expression of recombinant LRP4. (A) Flow cytofluorimetric analysis of parental untransfected HEK293T cells labeled with the rabbit anti-LRP4 antiserum (shaded area), compared to LRP4fl-HEK293T transfected cells (full line). (B) Precipitated supernatants from EGFP-HEK293T LRP4ecto-HEK293T cells were analyzed by anti-c-Myc immunoblotting; the band corresponding to the ecto-LRP4-myc tag fusion protein is indicated by arrow.

Following transfection with the pcDNA3.1-LRP4fl-myc/His plasmid, surface expression of LRP4 in HEK-293T cells could be easily detected by FACS analysis using rabbit anti-LRP4 antiserum (Fig 1A).

Cells transfected with pcDNA3.1-LRP4ecto-myc/His produced a soluble form of the extracellular domain, which could be precipitated from cell supernatants and detected by western blotting using the anti-c-Myc mAb (Fig 1B).

### FACS analysis of serum samples

**Fig 2:**
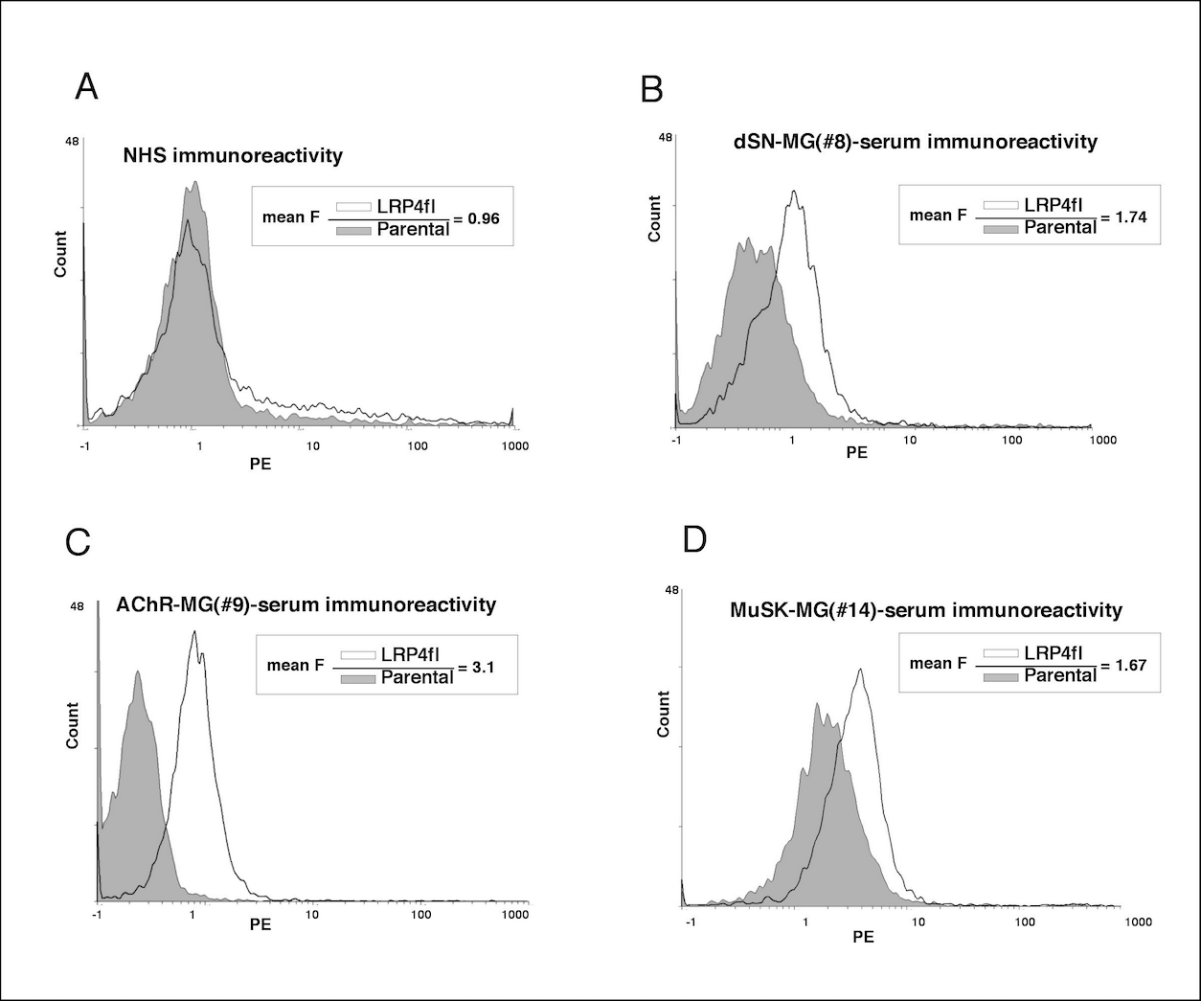
Anti-LRP4 detection by FACS analysis. Each of 101 MG s and 85 control sera was tested on both parental untransfected (shaded area) and on LRP4fl-HEK293T transfected cells (full line). None of the 45 NHS but only 14 MG sera showed a clear cut shift of mean fluorescence value with a ratio transfected/untransfected > 1.5. We show the immunoreactivity of one NHS, one dSN-MG (sample#8), one AChR-MG (sample#9) and one MuSK-MG serum (sample#14) (A,B,C,D respectively) as representative plots.

Serum samples from 101 MG patients, from 45 HBD and from 40 OND patients were tested on both parental un-transfected and LRP4fl-transfected HEK293T cells. While none of the normal human sera (NHS) showed immunoreactivity, 14/101 patients sera showed a clear-cut shift of mean fluorescence intensity (MFI transfected/MFI untransfected ratio > 1,5) (Fig 2). Among these, 8 were from 55 dSN-MG patients (14.5%), 3 from 23 AChR-MG (13%) and 3 from 23 MuSK-MG patients (13%) (Table 1). Only one out of the additional 40 OND (1/11 PM) showed anti LRP4 immunoreactivity.

**Table 1:** Anti-LRP4 immunoreactivity in 101 Italian myasthenic patients (MG) and controls.

Patients	Number	Anti-LRP4 immunoreactivity	%	(95% CI*)
**Total MG**	101	14	13.8	8–22
**double seronegative**	55/101	8	14.5	7.5–26.1
**AChR positive**	23/101	3	13	4.5–32.1
**MuSK positive**	23/101	3	13	4.5–32.1
**HBD**	45	0	0	0–7.9
**PM**	11	1	9	1.6–37.7
**MM**	10	0	0	0–27.7
**MS**	9	0	0	0–29.9
**ALS**	10	0	0	0–27.7

*:CI: Confidence Interval

### Immunoprecipitation and Blotting analysis

**Fig 3:**
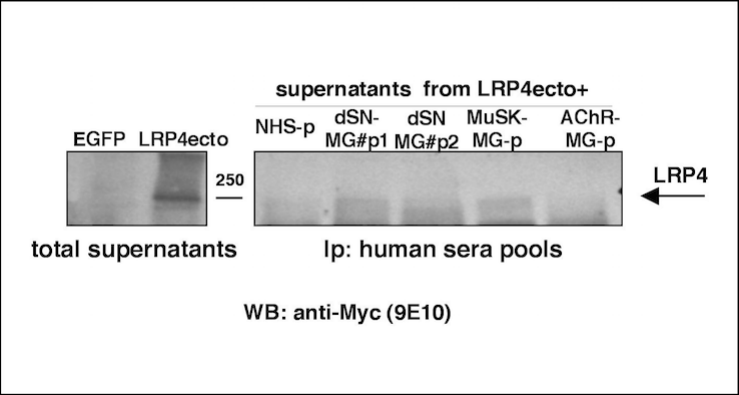
Anti-LRP4 detection by immunoprecipitation of pools of sera. Supernatants from LRP4ecto HEK293T cells were immune-precipitated with the indicated pools (p) of sera that scored positive at FACS analysis: pool#1 and #2 from dSN-MG, Musk-MG pool and AChR-MG pool. Immuno-complexes were subdued to western blotting and probed with anti-c-Myc. Aliquots of total supernatants from EGFP-HEK293T and from LRP4ecto HEK293T cells were blotted alongside with immune-precipitates as negative and positive control, respectively. The specific, uppermost band is pointed by the arrow.

We were not able to immunoprecipitate the soluble, extracellular domain of LRP4 with sera from individual patients. When, to increase sensitivity, we used pools of those sera that were positive at FACS analysis, the MuSK-MG pool gave the best positive result; the two dSN-MG pools were also able, although to a different extent (pool #1 > pool #2), to immunoprecipitate the antigen; instead the AChR pool did not precipitate any band of the expected size as assessed by anti-myc tag immunoblotting (Fig 3).

### Correlation with clinical characteristics

The whole MG population included 68 women and 33 men (ratio F:M: 2.06). The 14 LRP4 positive (pos) patients showed an increased female preponderance, 11 women and 3 men, when compared to the 87 LRP4 negative (neg) patients, 57 women and 30 men: ratio F:M of 3.6 versus 1,9. When MG patients are subdivided in early onset (EO), according to age of onset ≤ 50 years, and late onset (LO), the whole MG population included 71 EO and 30 LO (ratio EO:LO: 2.3). We found that the LRP4-pos patients were younger, 11 EO and 3 LO, when compared to the LRP4-neg patients, 60 EO and 27 LO: ratio EO:LO of 3.6 versus 2.22.

Considering only the 55 dSN-MG group (Table 2), there were 39 women and 16 men (ratio F:M: 2.4). Among these, the 8 dSN/LRP4-pos patients displayed a female predominance including 7 women and 1 men while the 47 dSN/LRP4-neg patients included 32 women and 15 men: ratio F:M of 7 versus 2.1. Considering age at onset, the whole dSN-MG group included 41 EO and 14 LO (ratio EO:LO: 2.9). Also in this case we found that the 8 dSN/LRP4-pos patients displayed a younger age at onset predominance, including 7 EO and 1 LO while the 47 dSN/LRP4-neg patients including 34 EO and 13 LO: ratio EO:LO of 7 versus 2.6.

**Table 2:** Clinical correlates with and without anti-LRP4 antibodies in double seronegative myasthenic patients.

Double seronegative myasthenic patients		LRP4-positive (n°)	LRP4-negative (n°)	Total (n°)
**Patients**		8	47	55
**Gender**	Female (F)	7	32	39
	Males (M)	1	15	16
**Age at onset**	Early (EO)	7	34	41
	Late (LO)	1	13	14
**Maximum MGFA**	I, IIA, IIB	6	22	28
	IIIA, IIIB, IVA	2	20	22
	IVB, V	0	5	5
**Current therapy**	No therapy or symptomatic	1	13	14
	Corticosteroid < 25mg/die	2	13	15
	Corticosteroid > 25mg/die or other immunosuppressors	5	21	26
**Thymoma**	Yes	1	2	3
	No	7	45	52
**Therapy at the moment of sample collection**	Yes	0	33	33
	No	8	14	22

Because of the small size of the population, the above differences are not statistically significant.

The 8 dSN/LRP4-pos patients were without therapy at the moment of sample collection and showed a less severe disease (Table 2): mild disease (class I, IIA, IIB) in 6 cases and moderate disease (class IIIA, IIIB, IVA) in 2 cases, while no patient showed a severe disease (grade IVB, V). No correlation with thymic pathology was found; only one patient displayed thymoma.

## Discussion

Low-density lipoprotein receptor-related protein 4 (LRP4) has recently been identified as the agrin receptor and has emerged as a third autoantigen in MG patients.

The detection of specific autoantibodies is a serological hallmark in the diagnosis of autoimmune diseases. We have developed a CBA in which the transfected autoantigen is expressed on the surface of HEK293T cells or shedded in the medium as soluble protein. In the present study, FACS analysis of membrane-directed immunoreactivity proved to be far more sensitive than immunoprecipitation/immunoblotting of soluble LRP4, although we cannot rule out the possibility that this difference is simply due to the low affinity of anti-LRP4 antibodies for protein-A sepharose, employed to precipitate the immune-complexes. Further, the soluble extracellular domain of LRP4 can undergo conformational changes and no longer be recognized by antibodies. Using FACS assay, we analyzed sera from 101 MG patients for the presence of LRP4 abs.

Previous studies have identified LRP4 abs in 2–45% of dSN- MG patients of different ethnicities and countries of origin; in these reports, the co-occurrence of either AChR or MuSK abs was found in some cases [15,17,18]. The different positivity rates could be ascribed to different methods: Higuchi and coworkers used an immunoprecipitation technique and a recombinant fusion protein made of the extracellular LRP4 domain fused to luciferase as antigen [15]; Pevzner and coworkers used indirect immunofluorescence on LRP4-transfected cells [17], while Zhang and coworkers used an ELISA with the recombinant extracellular domain of LRP4 [18]. Zisimopoulou and coworkers screened a large dSN-MG sample collection, consisting of 635 sera from 10 countries including Italy [29]. Through a CBA based on human LRP4 transfected-HEK293 cells, they found a positivity rate of 18.7%, with variations among different populations (range 7–32.7%). Our results are in agreement with this last one: using a similar CBA, but with a different detection system, we found a 14.3% frequency of positive results in an Italian series of 55 dSN-MG. Noteworthy, all the 8 LRP4-pos patients were without therapy at the time of sample collection, suggesting that anti-LRP4 antibodies may be particularly sensitive to the action of the therapy; if so, their frequency in the dSN-MG group could actually be underestimated.

Interestingly, Zisimopoulou identified double positive (AChR/LRP4 and MuSK/LRP4) sera among those from other countries, but not from Italy. In contrast we detected double positive sera in our series: anti-LRP4 antibodies were found in 3/23 anti-AChR positive and in 3/23 anti-MuSK positive sera.

The detection of anti-LRP4 antibodies in patients' sera represents a significant advance in the understanding and in the diagnosis of dSN-MG. In the most updated classification, Berrih-Aknin and coworkers reported that approximately 90% of the total MG patients show anti-AChR antibodies, that 5% of MG patients have anti-MuSK antibodies and ascribe anti-LRP4 antibodies to the 2% of the remaining 5% dSN-MG (range 12–50% of seronegative population) [4]. In keeping with this classification, we here report anti-LRP4 antibodies in 14,3% of a population of 55 dSN-MG; thus, our contribution confirms the importance of anti-LRP4 detection as a useful diagnostic tool to decrease the percentage of MG (no longer “double” but “triple”) seronegative patients.

According to the presence of autoantibodies the MG forms are generally divided in different subgroups with specific clinical features. AChR-MG displays three main clinical pictures: a) the pure ocular form; b) the generalized EO form with female prevalence and follicular thymic hyperplasia; c) the generalized LO form with an equal ratio of female to men and frequent association with thymoma [30]. MuSK-MG patients are typically female, and have a severe form of the disease with frequently affected facial, bulbar and respiratory muscles, whereas ocular symptoms and thymic abnormalities are rare [6, 31]. The clinical phenotype of the patients presenting anti-LRP4 antibodies is not well defined. The most update classification reported a younger female prevalence mainly without association with thymic pathology [4]. Accordingly, albeit with the limitation of the small size of the screened population, we report a female prevalence with a mild form of disease in LRP4-dSN-MG patients.

Our results confirm anti-LRP4 antibodies as the third class of autoantibodies in MG as validated by recent literature [4, 29], although without association with a particular clinical picture. Differently from anti-AChR and anti-MuSK abs, that seem to be mutually exclusive, the present study confirm that anti-LRP4 abs could associate with anti-AChR or anti-MuSK abs.

During the current year new antibodies were indicated as putative auto-abs in MG: anti-agrin and anti-cortactin (a protein that acts downstream from agrin/MuSK/LRP4, promoting AChR clustering) [32–34]. These new data strengthen the hypothesis that auto-abs interfering with agrin/LRP4/MuSK signaling at the NMJ can induce MG. However, in our opinion, anti-agrin and anti-cortactin autoantibodies do not improve the diagnostic performance of MG. In fact, as reported by Gasperi and coworkers, anti-agrin abs were not found in any double sero-negative-MG sera but only in 5/54 MG-patients, who were also single positive for anti-AChR or anti-MuSK auto-abs or double-seropositive for anti-MuSK/LRP4 abs [33]. Anti-cortactin abs were identified by Gallardo and coworkers as potential autoantibodies in seronegative MG but they were found in 12,5% of patients with other autoimmune disorders and also in 5,2% of healthy controls [34].

LRP4 auto-abs seem to be related to MG disease: only one out of 40 OND patients (1/11 PM) showed anti LRP4 immunoreactivity.

With a frequency of 0% in our NHS group, we confirm Zisimopoulou’s data that found anti-LRP4 antibodies in none of NHS series (0%). Nevertheless, while this manuscript was in preparation, Tzartos and coworkers reported a high and persistent frequency of anti-LRP4 abs in the sera (23.4%) and cerebrospinal fluid of patients with amyotrophic lateral sclerosis suggesting that LRP4 autoimmune reactivity may be more widely related to damage of LRP4 expressing tissues such as NMJ but also motor neurons and brain. Anti-LRP4 abs in ALS could represent the autoimmune component suggested for many years with a hypothetical (currently not shown) pathogenic role in the neurodegenerative process [35]. In our 40 OND sera we included also 10 ALS but we didn’t found anti-LRP4 antibodies in this series. Moreover, these new data on the occurrence of anti-LRP4 abs in ALS should be confirmed and validated by further studies.

In conclusion, based on ours and others’ results, we suggest that, in presence of a characteristic clinical picture, pharmacological response and/or electromyography suggestive for MG, without anti-AChR or anti-MuSK antibodies, the anti-LRP4 detection may represent an additional element in favor of the diagnosis. However, due to their limited specificity for MG and in spite of the proven pathogenic role in experimental models, anti-LRP4 alone cannot be considered quite as indicative as anti-AChR and anti-MuSK abs in the diagnostic algorithm for the disease. Moreover, further validated and standardized tests are critically needed to make the recognition and quantitation of LRP4 immunoreactivity more reliable and useful in the clinical management of MG patients.

## Supporting Information

S1 Dataset The anonymous data set of MG patients. (DOCX)

S2 Dataset The anonymous data set of controls. (DOCX)

S1 Fig Cell based assay for LRP4 antibody detection. For immunofluorescence detection of LRP4fl-transfected cells, un-transfected (A) and transfected (B) HEK293T cells were fixed with methanol, washed three times with cold PBS, blocked for10 min at room temperature with PBS/BSA 5% and incubated at 4°C with LRP4-positive patient serum at 1:100 dilution in PBS/BSA 0.1% (PBS-BSA), for 30 min. FITC conjugated anti-human IgG goat antiserum (AXA Diagnostics, Italy) were added at a 1:100 dilution in PBS-BSA and the samples incubated for 30 min at 4°C. Cells were examined on a Nikon Eclipse E600 fluorescence microscope.
(TIF)
