# Supplementary material for: Flow Cytofluorimetric Analysis of Anti-LRP4 (LDL Receptor-Related Protein 4) Autoantibodies in Italian Patients with Myasthenia Gravis
Source: PLoS One. 2015 Aug 18;10(8):e0135378. doi: 10.1371/journal.pone.0135378 (PMC4540439; doi:10.1371/journal.pone.0135378)
Supplement: S1 Dataset — (DOCX) [file pone.0135378.s001.docx]

**[S1 Table.](http://journals.plos.org/plosone/article/asset?unique&id=info:doi/10.1371/journal.pone.0129159.s001)The anonymous data set of MG patients**

| **Number** | **Gender** | **anti-AChR Abs** | **Anti-MuSK Abs** | **anti-LRP4 Abs** | **Disease** |
| --- | --- | --- | --- | --- | --- |
| 1 | M | neg | neg | neg | EOMG |
| 2 | F | neg | neg | neg | EOMG |
| 3 | F | neg | neg | **pos** | EOMG |
| 4 | F | neg | neg | **pos** | EOMG |
| 5 | F | neg | neg | neg | EOMG |
| 6 | F | neg | neg | neg | LOMG |
| 7 | F | neg | neg | neg | EOMG |
| 8 | F | neg | neg | **pos** | EOMG |
| 9 | M | 12.5 | neg | **pos** | EOMG |
| 10 | F | 2.3 | neg | neg | LOMG |
| 11 | M | 2.9 | neg | neg | EOMG |
| 12 | M | 17.1 | neg | neg | EOMG |
| 13 | M | 20.0 | neg | neg | EOMG |
| 14 | F | neg | 1 | **pos** | LOMG |
| 15 | F | neg | 1.22 | neg | EOMG |
| 16 | F | neg | 1.51 | neg | LOMG |
| 17 | F | neg | 0.83 | neg | LOMG |
| 18 | F | neg | 1.75 | neg | EOMG |
| 19 | M | 3.9 | neg | neg | LOMG |
| 20 | M | 1.1 | neg | **pos** | EOMG |
| 21 | F | 2.4 | neg | neg | LOMG |
| 22 | M | 2.4 | neg | neg | EOMG |
| 23 | F | 20.0 | neg | neg | EOMG |
| 24 | F | neg | neg | neg | EOMG |
| 25 | M | neg | neg | neg | EOMG |
| 26 | M | neg | neg | neg | LOMG |
| 27 | F | neg | neg | neg | EOMG |
| 28 | M | neg | neg | neg | EOMG |
| 29 | F | neg | neg | neg | LOMG |
| 30 | F | neg | neg | neg | EOMG |
| 31 | F | neg | neg | neg | EOMG |
| 32 | M | neg | neg | neg | EOMG |
| 33 | M | neg | neg | neg | LOMG |
| 34 | F | neg | neg | neg | EOMG |
| 35 | F | neg | neg | **pos** | EOMG |
| 36 | F | neg | neg | neg | LOMG |
| 37 | F | neg | neg | neg | EOMG |
| 38 | M | neg | neg | **pos** | EOMG |
| 39 | F | neg | neg | neg | LOMG |
| 40 | F | neg | neg | neg | LOMG |
| 41 | F | neg | neg | neg | EOMG |
| 42 | F | neg | neg | **pos** | EOMG |
| 43 | F | neg | neg | neg | EOMG |
| 44 | F | neg | neg | neg | EOMG |
| 45 | F | neg | neg | neg | EOMG |
| 46 | F | neg | neg | neg | EOMG |
| 47 | F | neg | neg | neg | EOMG |
| 48 | F | neg | neg | neg | EOMG |
| 49 | F | neg | neg | neg | EOMG |
| 50 | F | neg | neg | neg | EOMG |
| 51 | M | neg | neg | neg | EOMG |
| 52 | F | neg | neg | neg | EOMG |
| 53 | M | neg | neg | neg | LOMG |
| 54 | F | neg | neg | neg | EOMG |
| 55 | F | neg | neg | neg | EOMG |
| 56 | F | neg | neg | neg | LOMG |
| 57 | M | neg | neg | neg | EOMG |
| 58 | M | neg | neg | neg | EOMG |
| 59 | M | neg | neg | neg | LOMG |
| 60 | M | neg | neg | neg | EOMG |
| 61 | F | neg | neg | neg | LOMG |
| 62 | F | neg | neg | neg | EOMG |
| 63 | F | neg | neg | **pos** | LOMG |
| 64 | M | neg | neg | neg | EOMG |
| 65 | F | neg | neg | neg | EOMG |
| 66 | F | neg | neg | neg | LOMG |
| 67 | F | neg | neg | **pos** | EOMG |
| 68 | M | neg | neg | neg | LOMG |
| 69 | M | neg | neg | neg | EOMG |
| 70 | F | neg | neg | neg | EOMG |
| 71 | F | 8.3 | neg | neg | EOMG |
| 72 | F | 8.0 | neg | neg | EOMG |
| 73 | F | >17.5 | neg | neg | EOMG |
| 74 | F | 7.2 | neg | neg | EOMG |
| 75 | M | >11.3 | neg | neg | LOMG |
| 76 | F | >12.6 | neg | neg | EOMG |
| 77 | F | 9.8 | neg | neg | LOMG |
| 78 | F | neg | 1.3 | neg | EOMG |
| 79 | F | neg | 1.23 | neg | EOMG |
| 80 | M | 1.3 | neg | neg | LOMG |
| 81 | F | neg | 9.4 | neg | LOMG |
| 82 | M | neg | pos | neg | LOMG |
| 83 | F | neg | 1.67 | **pos** | EOMG |
| 84 | F | neg | 1.38 | neg | EOMG |
| 85 | F | neg | 1.40 | **pos** | LOMG |
| 86 | F | 2.5 | neg | neg | LOMG |
| 87 | M | 3.6 | neg | neg | EOMG |
| 88 | M | 8.9 | neg | neg | LOMG |
| 89 | M | 6.7 | neg | neg | LOMG |
| 90 | M | 9.7 | neg | neg | LOMG |
| 91 | F | 3.7 | neg | **pos** | EOMG |
| 92 | F | neg | 0.70 | neg | EOMG |
| 93 | F | neg | 1.1 | neg | EOMG |
| 94 | M | neg | 1.1 | neg | EOMG |
| 95 | F | neg | pos | neg | EOMG |
| 96 | F | neg | pos | neg | EOMG |
| 97 | M | neg | pos | neg | EOMG |
| 98 | F | neg | 11.59 | neg | EOMG |
| 99 | M | neg | pos | neg | EOMG |
| 100 | F | neg | pos | neg | EOMG |
| 101 | F | neg | pos | neg | EOMG |

Number: progressive number of the myasthenic patients in the table

Gender: M: male; F: female

anti-AChR Abs: the value is positive when > 0.45 nmoles/l; neg: < 0.45 nmoles/l

anti-MuSK Abs: the value is positive when > 0.05 nmoles/l; neg: < 0.05 nmoles/l

anti-LRP4 Abs: the value is positive when the ratio between the LRP4fl-mean-F and the corresponding parental-mean-F was >1.5; negative: neg: < 1.5

Disease: Myasthenia Gravis was divided according to age at onset, EOMG: age at onset ≤ 50 years; LOMG: age at onset > 50 years.
