# Supplementary material for: Flow Cytofluorimetric Analysis of Anti-LRP4 (LDL Receptor-Related Protein 4) Autoantibodies in Italian Patients with Myasthenia Gravis
Source: PLoS One. 2015 Aug 18;10(8):e0135378. doi: 10.1371/journal.pone.0135378 (PMC4540439; doi:10.1371/journal.pone.0135378)
Supplement: S2 Dataset — (DOCX) [file pone.0135378.s002.docx]

**[S2 Table.](http://journals.plos.org/plosone/article/asset?unique&id=info:doi/10.1371/journal.pone.0129159.s001)The anonymous data set of controls.**

| **Number** | **Gender** | **Age** | **anti-LRP4 Abs** | **Disease** |
| --- | --- | --- | --- | --- |
| 1 | F | 42 | neg | HBD |
| 2 | F | 44 | neg | HBD |
| 3 | F | 33 | neg | HBD |
| 4 | M | 34 | neg | HBD |
| 5 | F | 64 | neg | HBD |
| 6 | F | 61 | neg | HBD |
| 7 | M | 46 | neg | HBD |
| 8 | F | 50 | neg | HBD |
| 9 | F | 25 | neg | HBD |
| 10 | F | 54 | neg | HBD |
| 11 | M | 53 | neg | HBD |
| 12 | F | 29 | neg | HBD |
| 13 | F | 48 | neg | HBD |
| 14 | F | 61 | neg | HBD |
| 15 | F | 30 | neg | HBD |
| 16 | M | 82 | neg | HBD |
| 17 | M | 45 | neg | HBD |
| 18 | M | 36 | neg | HBD |
| 19 | F | 39 | neg | HBD |
| 20 | F | 27 | neg | HBD |
| 21 | F | 39 | neg | HBD |
| 22 | F | 48 | neg | HBD |
| 23 | F | 41 | neg | HBD |
| 24 | F | 26 | neg | HBD |
| 25 | F | 23 | neg | HBD |
| 26 | M | 24 | neg | HBD |
| 27 | M | 50 | neg | HBD |
| 28 | F | 58 | neg | HBD |
| 29 | F | 43 | neg | HBD |
| 30 | F | 50 | neg | HBD |
| 31 | F | 37 | neg | HBD |
| 32 | M | 42 | neg | HBD |
| 33 | F | 81 | neg | HBD |
| 34 | F | 53 | neg | HBD |
| 35 | M | 69 | neg | HBD |
| 36 | M | 50 | neg | HBD |
| 37 | F | 51 | neg | HBD |
| 38 | F | 48 | neg | HBD |
| 39 | M | 58 | neg | HBD |
| 40 | M | 55 | neg | HBD |
| 41 | M | 48 | neg | HBD |
| 42 | F | 47 | neg | HBD |
| 43 | F | 49 | neg | HBD |
| 44 | M | 53 | neg | HBD |
| 45 | F | 56 | neg | HBD |
| 46 | F | 19 | neg | PM |
| 47 | M | 60 | neg | PM |
| 48 | F | 61 | neg | PM |
| 49 | M | 57 | neg | PM |
| 50 | M | 64 | neg | PM |
| 51 | F | 58 | **pos** | PM |
| 52 | F | 55 | neg | PM |
| 53 | F | 47 | neg | PM |
| 54 | F | 48 | neg | PM |
| 55 | F | 22 | neg | PM |
| 56 | F | 61 | neg | PM |
| 57 | M | 31 | neg | MM |
| 58 | F | 18 | neg | MM |
| 59 | M | 20 | neg | MM |
| 60 | F | 18 | neg | MM |
| 61 | F | 22 | neg | MM |
| 62 | M | 40 | neg | MM |
| 63 | F | 42 | neg | MM |
| 64 | F | 23 | neg | MM |
| 65 | M | 41 | neg | MM |
| 66 | F | 34 | neg | MM |
| 67 | F | 25 | neg | MS |
| 68 | F | 35 | neg | MS |
| 69 | M | 22 | neg | MS |
| 70 | F | 41 | neg | MS |
| 71 | F | 50 | neg | MS |
| 72 | F | 27 | neg | MS |
| 73 | M | 30 | neg | MS |
| 74 | F | 24 | neg | MS |
| 75 | M | 29 | neg | MS |
| 76 | M | 40 | neg | ALS |
| 77 | F | 58 | neg | ALS |
| 78 | M | 51 | neg | ALS |
| 79 | F | 59 | neg | ALS |
| 80 | F | 55 | neg | ALS |
| 81 | F | 45 | neg | ALS |
| 82 | M | 59 | neg | ALS |
| 83 | M | 55 | neg | ALS |
| 84 | M | 62 | neg | ALS |
| 85 | M | 63 | neg | ALS |

Legend:

Number : progressive number of the patients or controls in the table

Gender: M: male; F: female

Age: years at the blood collection

Anti-LRP4 Abs: the value is positive when the ratio between the LRP4fl-mean-F and the corresponding parental-mean-F was >1.5; negative; neg: < 1.5

DiseaseHBD: healthy blood donors; PM: polymyositis; MM: mitochondrial myopathy; MS: Multiple Sclerosis; ; ALS: amyotrophic lateral sclerosis.
