# Supplementary figures and images for: Flow Cytofluorimetric Analysis of Anti-LRP4 (LDL Receptor-Related Protein 4) Autoantibodies in Italian Patients with Myasthenia Gravis
Source: PLoS One. 2015 Aug 18;10(8):e0135378. doi: 10.1371/journal.pone.0135378 (PMC4540439; doi:10.1371/journal.pone.0135378)

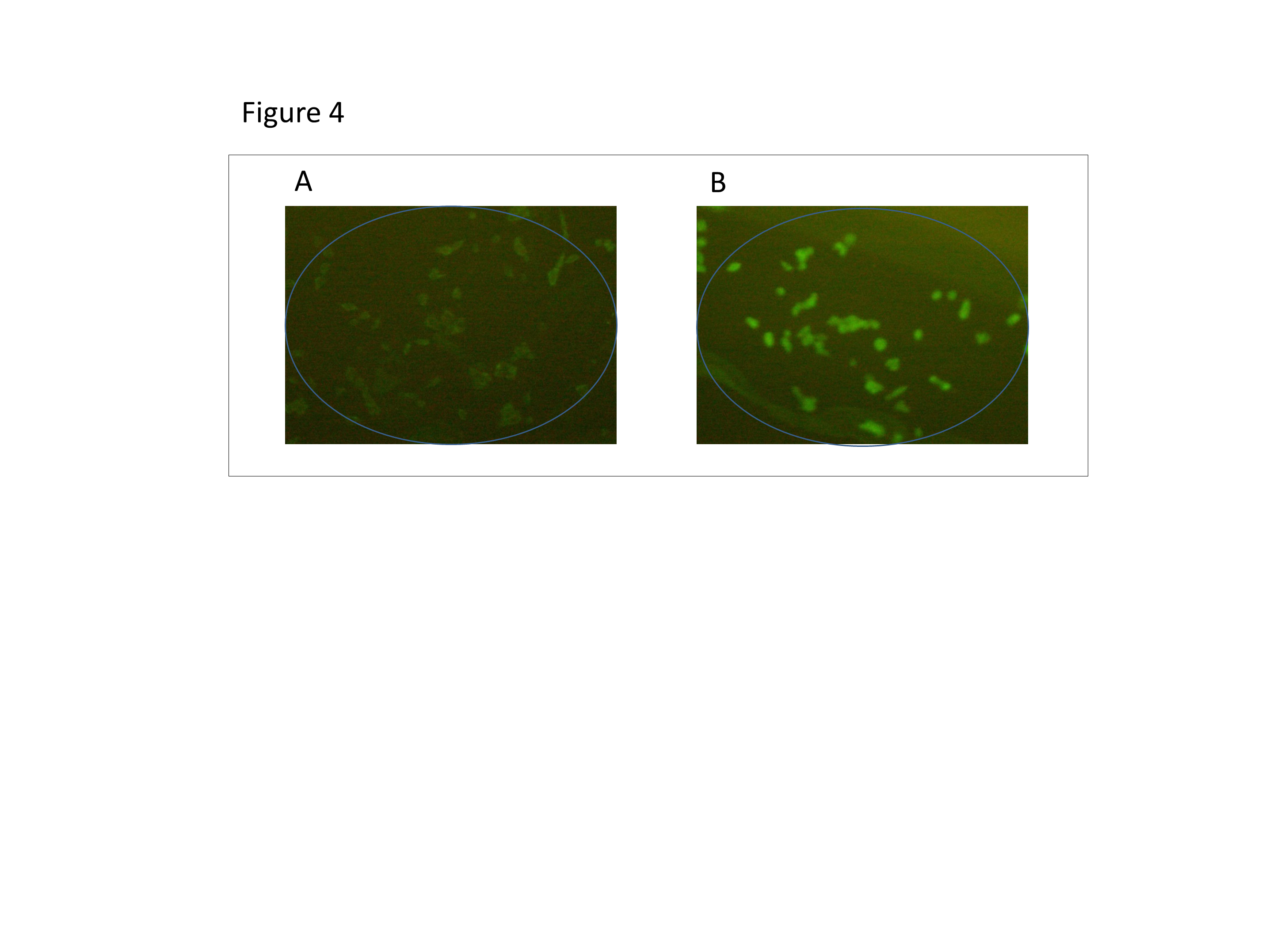

Supplement: S1 Fig — For immunofluorescence detection of LRP4fl-transfected cells, un-transfected (A) and transfected (B) HEK293T cells were fixed with methanol, washed three times with cold PBS, blocked for10 min at room temperature with PBS/BSA 5% and incubated at 4°C with LRP4-positive patient serum at 1:100 dilution in PBS/BSA 0.1% (PBS-BSA), for 30 min. FITC conjugated anti-human IgG goat antiserum (AXA Diagnostics, Italy) were added at a 1:100 dilution in PBS-BSA and the samples incubated for 30 min at 4°C. Cells were examined on a Nikon Eclipse E600 fluorescence microscope. (TIF) [file pone.0135378.s003.tif]
